# Full-Length Transcriptome Sequencing: An Insight Into the Dog Model of Heart Failure

**DOI:** 10.3389/fcvm.2021.712797

**Published:** 2021-12-16

**Authors:** Xiaoyan Liang, Zechen Bai, Feifei Wang, Yafan Han, Huaxin Sun, Jiasuoer Xiaokereti, Ling Zhang, Xianhui Zhou, Yanmei Lu, Baopeng Tang

**Affiliations:** ^1^Department of Pacing and Electrophysiology, The First Affiliated Hospital of Xinjiang Medical University, Ürümqi, China; ^2^Xinjiang Key Laboratory of Cardiac Electrophysiology and Cardiac Remodeling, The First Affiliated Hospital of Xinjiang Medical University, Ürümqi, China; ^3^Shenzhen Institute of Advanced Technology, Chinese Academy of Sciences (CAS), Shenzhen, China; ^4^Xinjiang First Aid Center, People's Hospital of Xinjiang Uygur Autonomous Region, Ürümqi, China

**Keywords:** heart failure, target genes, helper T cell differentiation, full-length transcriptome sequencing, transcript expression analysis

## Abstract

Heart failure (HF) leads to a progressive increase in morbidity and mortality rates. This study aimed to explore the transcriptional landscape during HF and identify differentially expressed transcripts (DETs) and alternative splicing events associated with HF. We generated a dog model of HF (*n* = 3) using right ventricular pacemaker implantation. We performed full-length transcriptome sequencing (based on nanopore platform) on the myocardial tissues and analyzed the transcripts using differential expression analysis and functional annotation methods [Gene Ontology (GO) and Kyoto Encyclopedia of Genes and Genomes (KEGG) analyses]. Additionally, we estimated the expression of the selected genes by quantitative real-time PCR (qRT-PCR) and detected the proportion of immune cells using flow cytometry. We found that increased B-type natriuretic peptide reduced ejection fraction, and apparent clinical signs were observed in the dog model of HF. We identified 67,458 transcripts using full-length transcriptome sequencing. A total of 785 DETs were obtained from the HF and control groups. These DETs were mainly enriched in the immune responses, especially Th1, Th2, and Th17 cell differentiation processes. Furthermore, flow cytometry results revealed that the proportion of Th1 and Th17 cells increased in patients with HF compared to controls, while the proportion of Th2 cells decreased. Differentially expressed genes in the HF and control groups associated with Th1, Th2, and Th17 cell differentiation were quantified using qRT-PCR. We also identified variable splicing events of sarcomere genes (e.g., *MYBPC3, TNNT2, TTN, FLNC*, and *TTNI3*). In addition, we detected 4,892 transcription factors and 406 lncRNAs associated with HF. Our analysis based on full-length transcript sequencing provided an analysis perspective in a dog model of HF, which is valuable for molecular research in an increasingly relevant large animal model of HF.

## Introduction

Heart failure (HF) is a rapidly developing cardiovascular disease associated with considerable morbidity, hospitalization, and mortality (1). The HF incidence, and the related economic burden, is predicted to escalate in the coming years due to age-related structural changes (2, 3). Recently, early diagnosis and intervention of HF have gained attention. B-type natural peptide (BNP) is a suitable biomarker for HF and is usually released in response to increased left ventricle load (4). Essentially, the complex pathophysiology of HF necessitates a multi-index analysis to establish effective diagnostic and prognostic biomarkers. Re-hospitalization of discharged patients with HF followed by mortality is common (5). Therefore, determining the pathological mechanisms that lead to HF is necessary for developing more effective HF therapies.

Transcriptome analysis in large animal model of HF is widely used in HF research (6, 7). In recent years, transcript data in HF model have been analyzed by the next-generation sequencing (NGS) or Affymetrix exon arrays (Santa Clara, CA, USA), such as prediction of alternative splicing (AS) events and lncRNAs (8, 9). However, these techniques had limitations in recognizing AS isoforms, homologous gene families, and complete and accurate assembly of transcripts because of short read data. With advances in sequencing technology, long-read sequences can be efficiently generated using techniques such as third-generation sequencing (TGS) (10). Advantages of TGS are summarized as follows: (1) it provides longer read data than NGS and have distinct error characteristics (11); (2) it increases the gene inheritance and functional diversity of the sequence by analyzing different transcript isoforms regulated by AS (12); and (3) it offers an alternative method for obtaining non-coding transcripts, ensures reliable qualitative analysis of AS transcripts, and improves transcriptomic annotation (13). At present, the TGS of high-frequency pacing for generating HF in dogs has not been established. Therefore, our study aimed to utilize TGS (based on nanopore platform) for the transcriptional profile analysis of myocardial tissue from a dog model of HF.

## Materials and Methods

### Production of a Rapid Pacing HF Dog Model

**Figure 1:**
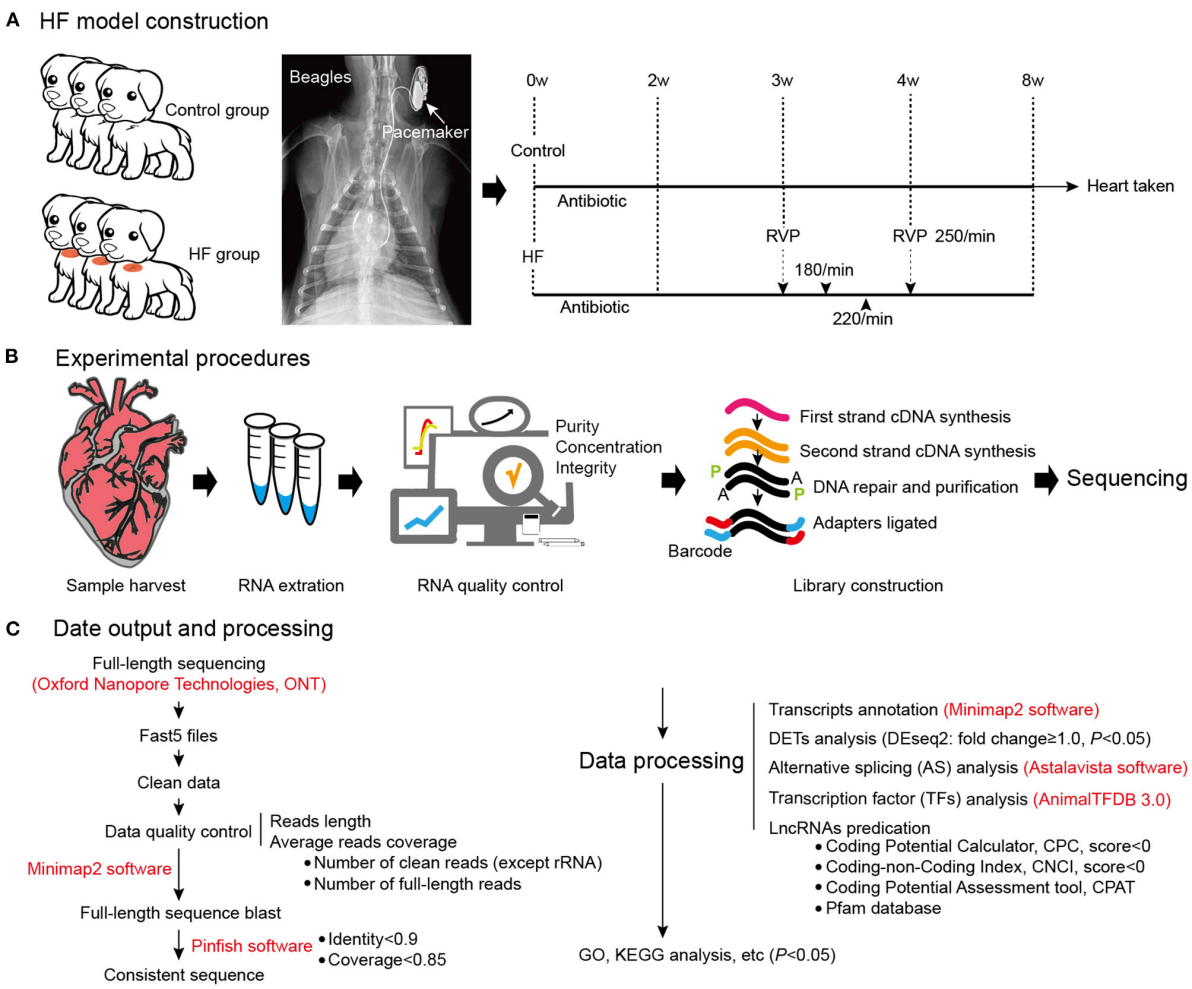
Dog model of HF construction and full-length transcriptome sequencing. **(A)** The workflow of HF model construction. The procedure for HF model manipulation at different times. **(B)** Experimental procedures. **(C)** Data output and processing. W, weeks; RVP, rapid ventricular pacing; HF, heart failure.

Six beagles were randomly divided into two groups: HF (*n* = 3) and control (*n* = 3). The control group dogs were subjected to a sham surgery involving cardiac pacemaker implantation, whereas right ventricular pacemaker implantation was used to produce the dog model of HF. Venous access was established according to a previously described procedure (10), and the ventilators were connected after intramuscular anesthesia injection using 3% pentobarbital. Subsequently, the right external jugular vein was exposed after separation from the subcutaneous tissue, and the introducer wire and vascular sheath were sequentially delivered intravenously. A pace-control spiral electrode was delivered with the aid of the ultrasound imagery, with the electrode tail connected to a modified human-pulse generator. Additionally, penicillin 3.2 million IU + 100 ml 0.9% NaCl intravenous infusion was administered intraoperatively and postoperatively. The dogs were observed under general conditions (temperature, 22–26°C; humidity, 50–70%, light-dark cycle, 12 h light, 12 h dark; unlimited food and water) and were subjected to cardiac ultrasound in their conscious state. After 3 weeks, the pacemaker was adjusted to 180 beats/min for 3 days; 220 beats/min for 3 days; and 250 beats/min for 3 weeks (Figure 1A). The extent of fibrosis in the myocardial tissues was detected by Masson's trichrome Staining, and Masson's positive regions were quantified using the ImageJ software (v1.8.0; National Institutes of Health, Bethesda, MD, USA). Meanwhile, the plasma concentrations of BNP and IL-17 were detected using an ELISA Kit (Jianglai, Shanghai, China). The right ventricular tissue morphology was observed using a transmission electron microscope (JEM-1220, JEOL Ltd., Tokyo, Japan), and images were captured using an OLYMPUS system (Olympus Soft Imaging Solutions, Morada G3, Japan).

### Ethics

Human and animal studies were reviewed and approved by the Ethics Committee of The First Affiliated Hospital of Xinjiang Medical University (K202105-18, CNU IACUC201902-K03) in accordance with the Declaration of Helsinki. Written informed consent was obtained from all participants.

### RNA Preparation and Full-Length Transcriptome Sequencing

Total RNA was extracted from the right ventricle myocardial tissues using an RNeasy Mini Kit (QIAGEN, Hilden, Germany) according to the instructions of the manufacturer. RNA purity was assessed using a NanoPhotometer® spectrophotometer (IMPLEN, Westlake Village, CA, USA), and the concentration was quantified using a Qubit Fluorometer (Thermo Fisher Scientific, Waltham, MA, USA). Full-length, first-strand cDNAs were enriched using 50 ng of the total RNA for each sample according to the cDNA-PCR Sequencing Kit (SQK-PCS109) according to the instructions of the manufacturer. Finally, the Oxford Nanopore Technologies (ONT, Oxford, UK) adaptor was used to ligate the PCR products using T4 DNA ligase [New England Biolabs (NEB), Ipswich, MA, USA]. The generated cDNA libraries were sequenced using PromethION platform.

### Nanopore Data Processing

Raw reads were normalized to a minimum average read-quality score of 7 and a minimum read-length of 500 bp. Full-length non-chimeric transcripts were determined by primer-searching at both ends of the reads. The barcode was as follows: 5′-TTTCTGTTGGTGCTGATATTGC and 3′- GAAGATAGAGCGACAGGCAAGT. Meanwhile, the transcripts were obtained by mapping the sequences to the reference genome using minimap2^1^, and redundant transcripts were excluded. The results of the transcript expression level quantification and full-length read differential analysis were mapped to the reference transcriptome sequence. The expression levels of the mapped reads were then estimated by reads per transcript for 10,000 mapped reads. Moreover, the counts per million (CPM) quantification technique was adopted for transcript expression measurements (14). Differential expression analysis of the HF and control samples was performed using the DESeq2 R package (1.6.3): http://www.bioconductor.org/packages/release/bioc/html/DESeq2 (15), with *p* < 0.05 representing differentially expressed transcripts (DETs). Furthermore, structural analysis of AS was performed. AS events were determined using the AStalavista tool:^2^ (16). In addition, animal transcription factors (TFs) were retrieved from the animal TF database. Four computational approaches were combined to sort the long non-coding RNAs (lncRNAs) from the transcripts: Coding Potential Calculator (CPC), Coding–Non-Coding Index (CNCI), Coding Potential Assessment Tool (CPAT), and Pfam. Statistical significance was set at *p* < 0.05. Eventually, the full-length transcriptome sequencing files were deposited in the SRA database (PRJNA731299).

### Functional Annotation Analysis

Gene Ontology (GO) and Kyoto Encyclopedia of Genes and Genomes (KEGG, https://www.kegg.jp/) pathways for the DETs were generated using the GOseq R package (17) and KOBAS^3^ software (18), respectively. The statistical enrichment of the differentially expressed genes (DEGs) in the KEGG pathway was then conducted using a false discovery rate (FDR) < 0.05. GO and KEGG pathways for AS, TFs, and lncRNA were generated using the Enrichr^4^ software. Statistical significance was set at *p* < 0.05.

### Flow Cytometry

Adults (≥18 years of age) with chronic HF (functional class II, III, or IV), a left ventricular ejection fraction (EF) of 45% or less, and BNP > 300 ng/L were eligible to participate in the study. Exclusion criteria were as follows: (1) acute renal insufficiency or chronic kidney disease stages III–IV; (2) hepatic insufficiency; (3) pregnant or lactating women; (4) patients with rheumatic immune system disease, severe pneumonia; and (5) malignant tumors (receiving active treatment) or other life-threatening diseases. Blood samples from patients with HF (*n* = 10) and controls (*n* = 10) were collected immediately after diagnosis. No significant difference was found in baseline demographics and clinical characteristics between patients with HF and controls (Table 1), indicating no selection bias (*p* ≥ 0.05). The cell surface antigens were stained according to the standard flow cytometry staining procedures using antibodies specific to CD4, CD196, and CD183 cells (BD Sciences, San Jose, CA, USA). The cells were then treated with red blood cell lysate (BD Sciences, San Jose, CA, USA) and washed twice with phosphate-buffered saline. Flow cytometry was performed using a BD LSR II flow cytometer and analyzed using the FlowJo v7 software (TreeStar, San Carlos, CA, USA).

**Table 1:** Baseline characteristics of patients with HF and controls.

**Variables**	**Controls** **(*n* = 10)**	**HF** **(*n* = 10)**	***P*-value**
Age (years)	63.3 ± 15.3	58.3 ± 17.8	0.596
Male	6 (60%)	6 (60%)	1.000
BMI (kg/m^2^)	25 ± 3.5	25 ± 2.2	>0.999
**Hamodynamics**			
Systolic blood pressure (mmHg)	113.8 ± 6.9	128.9 ± 21.7	0.05
Diastolic blood pressure (mmHg)	70.7 ± 6.4	72.1 ± 15.9	0.799
Left ventricle ejection fraction (%)	62.5 ± 1.9	36.6 ± 8.0	<0.0001
**Laboratory values**			
Total cholesterol (mmol/L)	3.9 ± 0.9	4.2 ± 1.0	0.560
Low-density lipoprotein (mmol/L)	2.3 ± 0.4	2.8 ± 0.7	0.08
High density lipoprotein (mmol/L)	l.l ± 0.5	0.8 ± 0.2	0.08
Triglycerides (mmol/L)	1.4 ± 0.7	1.5 ± 0.6	0.85
N-preBNP (ng/L)	128.2 (68.08–261.8)	3120.0 (1043.8–6172.5)	<0.001
Creatinine (μmmol/L)	70.0 ± 13.7	85.8 ± 28.8	0.13

*Values are mean ± SD, n (%) or median (interquartile range); HF, heart failure; BMI, body mass index*.

### Quantitative Real-Time PCR

Total RNA was extracted from the myocardial tissues of the HF and control groups using a TRIzol extraction kit (Invitrogen, Carlsbad, CA, USA). Reverse transcription of RNA into cDNA was then conducted using the First-Strand cDNA Synthesis SuperMix (Takara, Dalian, China). Quantitative real-time PCR (qRT-PCR) was performed using specific primers (Table 2) according to the SYBR Green PCR Kit (Invitrogen, Carlsbad, CA, USA) according to the protocol of the manufacturer. About 35 PCR cycles were used for the amplification. GAPDH mRNA expression level was used as an internal standard, and the results were analyzed using the 2^−ΔΔCt^ method.

**Table 2:** The primers used.

**Genes**	**Primers**
GAPDH	F: 5′-GCAAATTCCACGGCACAGTCAAG-3′
	R: 5′-ACAACATACTCAGCACCAGCATCAC-3′
JUN	F: 5′-AGAACTCGGACCTGCTCACCTC-3′
	R: 5′-GATGTGCCCGTTGCTGGACTG-3′
JAG2	F: 5'-GGTCGTCATGGCAGCTTCTTCC-3'
	R: 5'-GGCTCCTCTCCCGCTCTTTCC-3'
FOS	F: 5′-CCCGTAGTCACCTGTACTCCTAGC-3′
	R: 5′-GCTGCTGCCCTTGCGATGAG-3′
DLA-DMA	F: 5′-CGTTGAAGCCCCTGGAGTTTGG-3′
	R: 5′-ATGCCACCAGTTCACCGTCAATG-3′
DLA-DQB1	F: 5′-CAAGCCCTGTCACCGTGGAATG-3′
	R: 5′-CGAAGCCACCAATGCCACTCAG-3′
HLA-DRB1	F: 5′-CAAGCCCTGTCACCGTGGAATG-3′
	R: 5′-GAAGAGCAGACCCAGGACAAAGC-3′
DLA-DRA	F: 5′-ACCCATCAGGCGAGTTCATGTTTG-3′
	R: 5′-GCCACACCGTCTCCTTCTTTTCC-3′

### Statistical Analysis

Statistical significance between the two groups was analyzed by Student's *t*-test in SPSS 19.0 (IBM, USA). Data were recorded as the mean ± SD. Differences were considered significant at *p* < 0.05.

## Results

### Generation of the Experimental Rapid Pacing Dog Model

**Figure 2:**
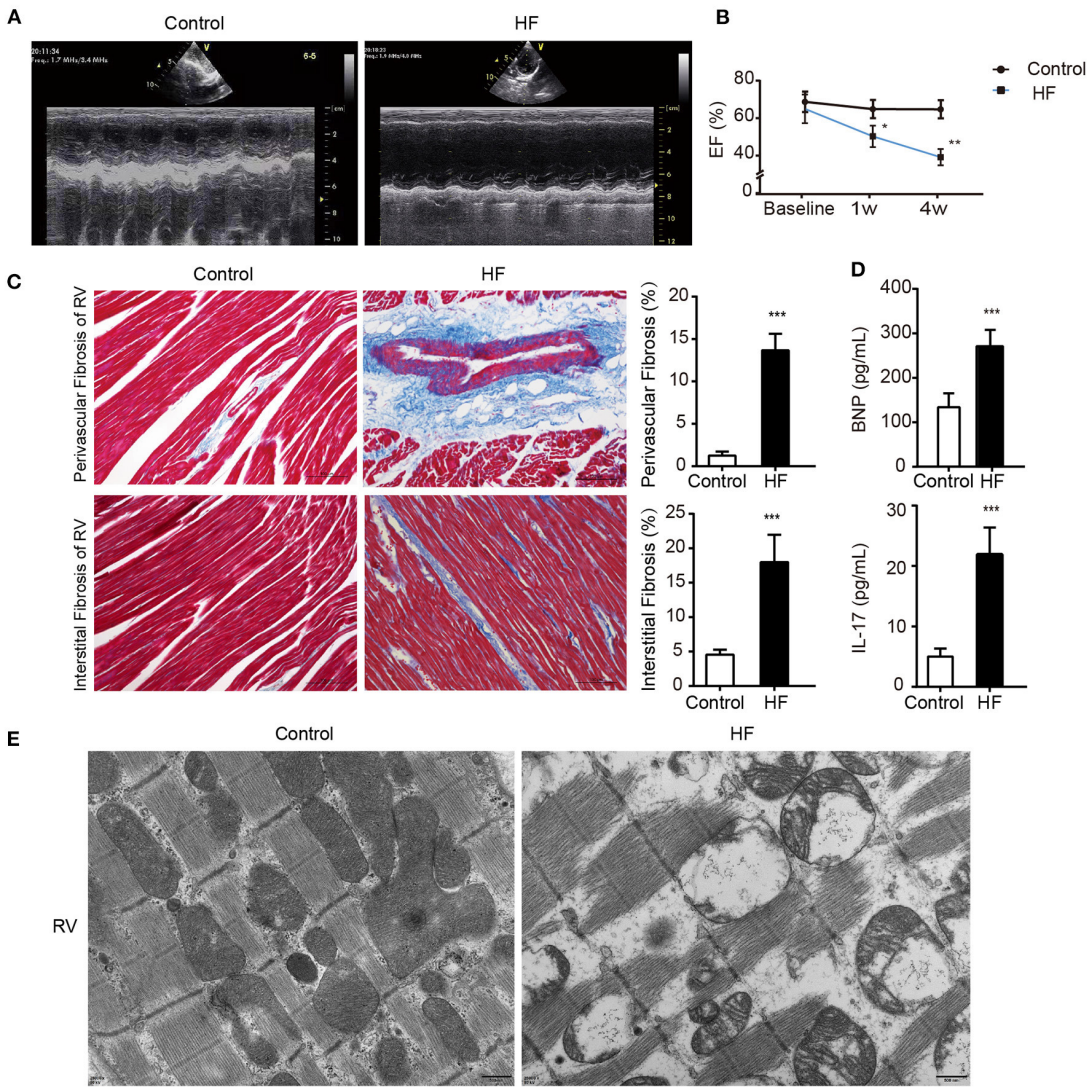
Successful establishment of HF dogs. **(A)** Echocardiography in HF and control groups. **(B)** The EF on the 1st and 4th week of HF. *p < 0.05, **p < 0.01. **(C)** Masson staining of the right ventricular myocardium of HF and control groups. **p < 0.01. Bar = 100 μm. **(D)** Levels of BNP and IL-17 in the plasma of HF and control groups. **p < 0.01. **(E)** The morphology of the right ventricular tissue under transmission electron microscopy. Bar = 500 nm. BNP, B-type natural peptide; EF, ejection fraction; HF, heart failure; RVP, right ventricle pacing. ***p <0.01.

weeks after the modeling surgery, the heart ultrasounds of the dogs showed normal cardiac function and proper electrode fixation (Figure 1). Heart ultrasounds detected a gradual decrease in the left ventricular EFof the HF group with a prolonged postoperative time (Figures 2A,B) compared to the control group (*p* < 0.05). The HF group had an EF of <45% after 4 weeks of rapid ventricular pacing (RVP). Masson's staining showed myocardial perivascular and interstitial fibrosis in the HF group (*p* < 0.01) (Figure 2C). Moreover, plasma BNP was significantly increased in the HF group (*p* < 0.01) (Figure 2D), indicating that the dog model of HF was successfully established. Meanwhile, IL-17, secreted by Th17 cells, increased in the HF group (*p* < 0.01) (Figure 2D). Additionally, sarcomere injuries were observed by transmission electron microscopy in the HF group (Figure 2E).

### Detection of DETs Associated With HF

**Figure 3:**
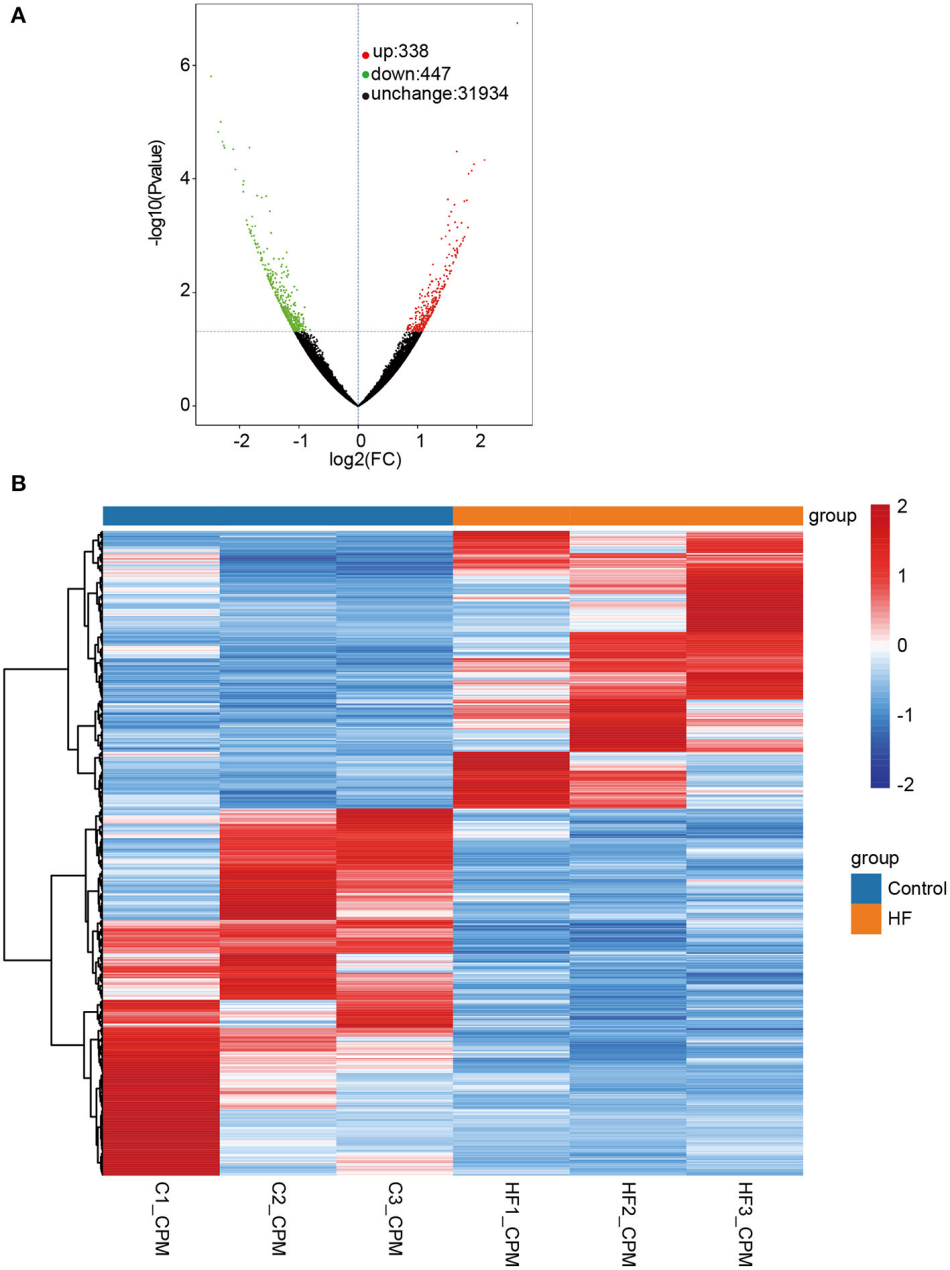
Differentially expressed transcripts (DETs) between HF dogs and controls. **(A)** Volcano plot of DETs. Green represents the downregulated DETs, red represents the upregulated DETs, and black represents the non-differential expression. **(B)** Heatmap of the DETs expression levels in HF dogs and controls. Red and blue represent up- and downregulated DETs, respectively. HF, heart failure; C, controls; FC, fold change.

The quality assessment of full-length transcriptome sequencing (based on nanopore platform) data in this study is shown in Supplementary Table 1. The number of full-length sequences obtained from each sample varied from 1,105,125 to 1,800,561 (Supplementary Table 2), and the average mapped rates were 91.31%. The results illustrated that the nanopore sequencing data were sufficient for subsequent analysis. A consensus isoform sequence was obtained by polishing the full-length sequence analysis for all consensus transcript sequences after alignment with the reference genome. Finally, 67,458 non-redundant transcripts and 23,734 annotated genes were identified. Among the 67,458 transcript sequences, the transcript expression ranged from log10 (CPM) = 0–2. We compared DETs for the HF and control groups, and a total of 785 DETs were obtained, including 338 upregulated and 447 downregulated DETs (Figure 3A and Supplementary File 1). In our study, up- and downregulated DETs were clustered (Figure 3B).

### Functional Annotation of DETs

**Figure 4:**
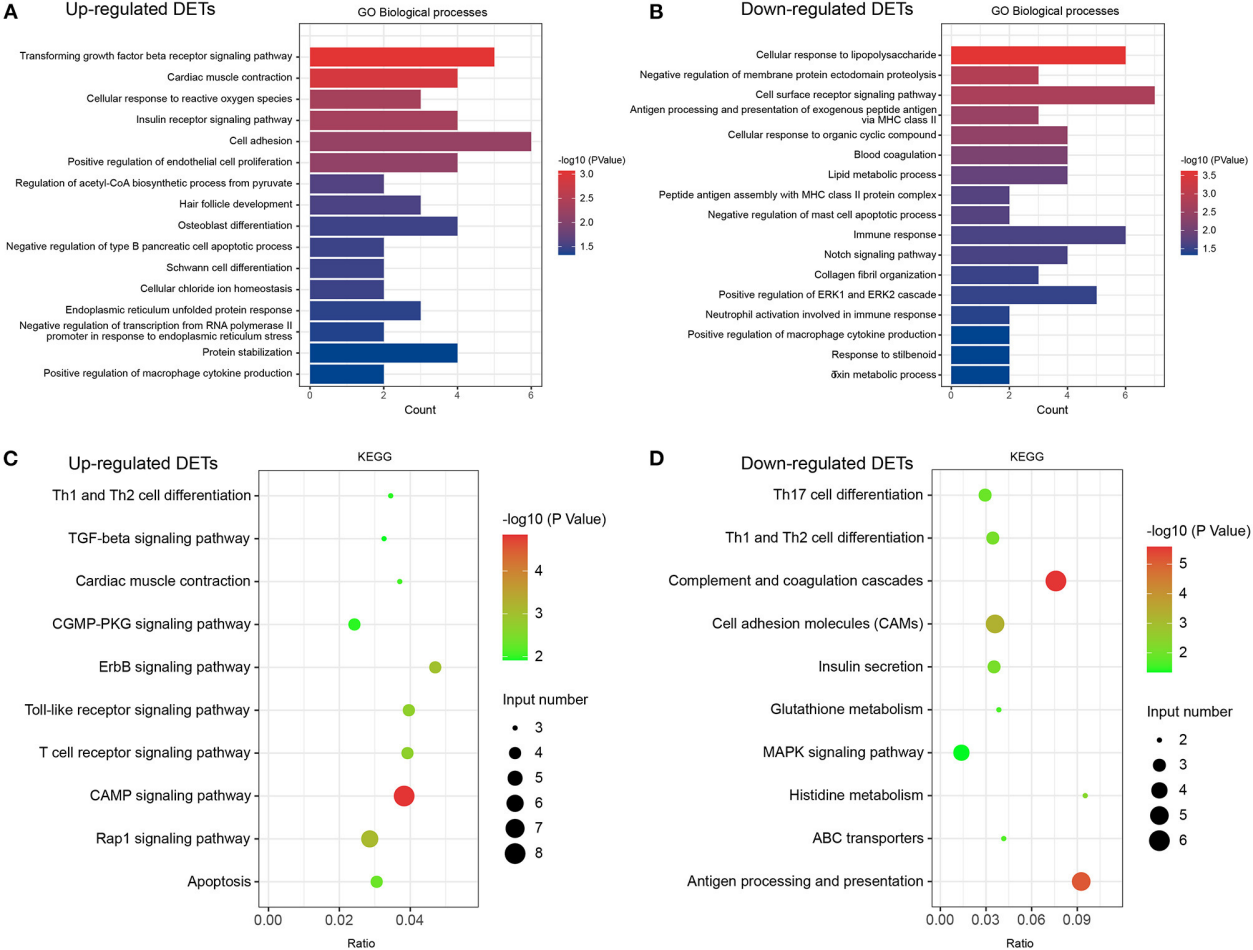
GO and KEGG analyses in DETs. **(A)** Classification of biological processes in GO annotation for upregulated DETs. **(B)** Category of biological processes in GO annotation for downregulated DETs. **(C)** KEGG annotation of upregulated DETs. **(D)** KEGG annotation of downregulated DETs. Only the significant pathways sections (*p* < 0.05) were presented in the figure. DETs, differentially expressed transcripts; GO, Gene ontology; KEGG, Kyoto Encyclopedia of Genes and Genomes.

We performed DET annotation analysis to determine the pathological mechanisms of HF. Upregulated DETs were annotated and assigned to a total of 16 biological processes of the GO enrichment analysis (Figure 4A), which included the transforming growth factor (TGF)-β receptor signaling pathway and cardiac muscle contraction. Conversely, the downregulated DETs were annotated and assigned to 17 biological processes (Figure 4B), including cellular responses to lipopolysaccharide and negative regulation of membrane protein ectodomain proteolysis. As shown in Figures 4C,D, up- and downregulated DETs were both enriched to Th-cell (Th1, Th2, and Th17) differentiation by the KEGG analysis. The TGF-β receptor signaling pathway, CGMP-PKG, apoptosis, and MAPK signaling pathways were highly enriched (*p* < 0.05) (Figures 4C,D). The cellular components and molecular functions of DETs are presented in Supplementary Figure 1. In addition, DEGs and corresponding GO/KEGG analysis were performed to provide an overview of transcriptome changes in the HF model (Supplementary Figure 2).

### Candidate Genes Associated With Th1, Th2, and Th17

**Figure 5:**
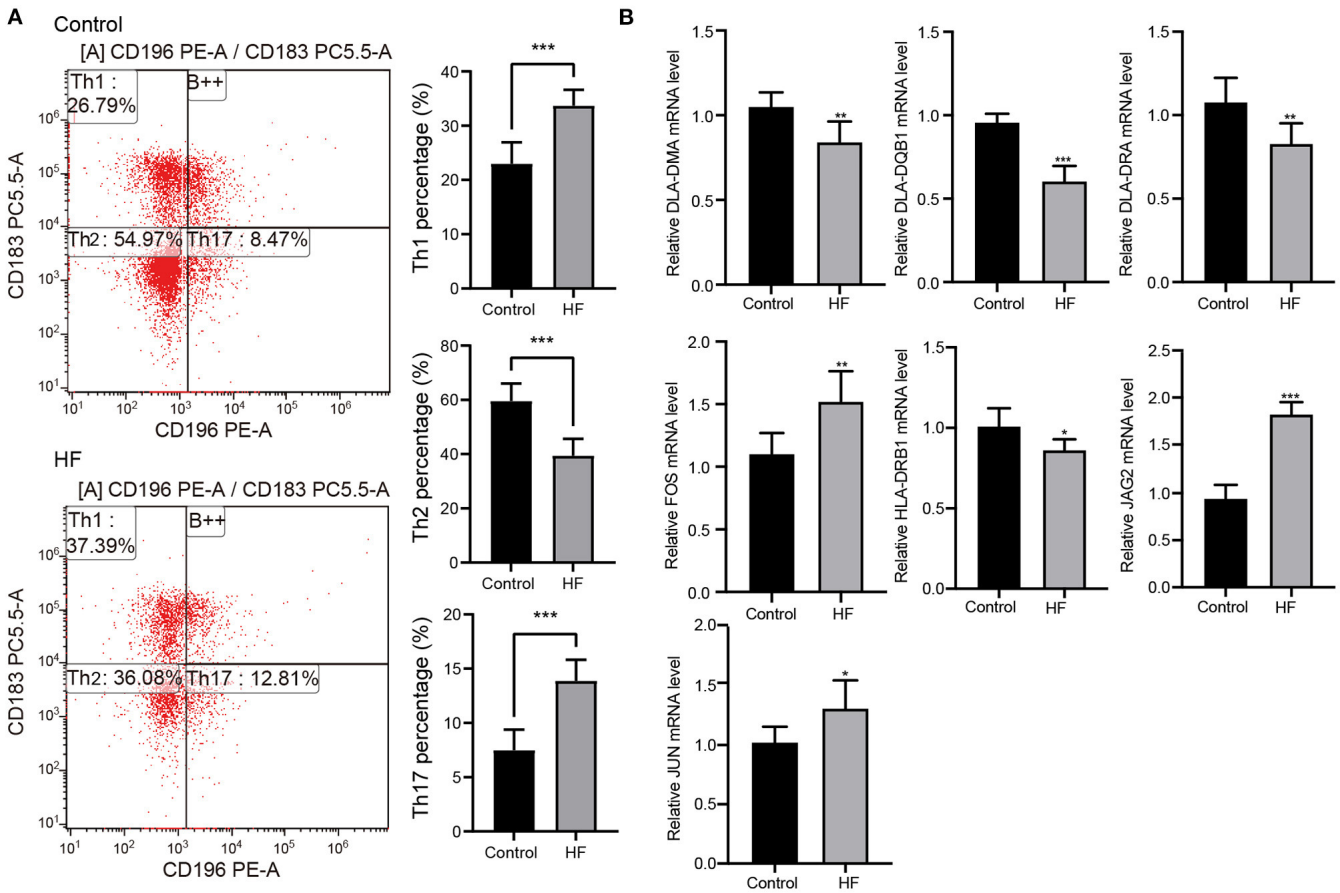
Flow cytometry and quantitative real-time PCR (qRT-PCR) detection. **(A)** The proportion change of Th1, Th2, and Th17 cells in patients with HF (*n* = 10) and controls (*n* = 10) detected using flow cytometry. ***p < 0.001. **(B)** Expression changes of the key genes in HF dogs and controls through qRT-PCR detection. *p < 0.05, **p < 0.01, ***p < 0.001. qRT-PCR, quantitative real-time PCR; HF, heart failure.

Flow cytometry was used to examine the levels of Th1, Th2, and Th17 cells to better understand the molecular mechanisms underlying myocardial failure (Figure 5A). Th1 and Th17 cells were more abundant in patients with HF (*p* < 0.001), and the proportion of Th2 cells decreased (*p* < 0.001). In addition, seven DETs (DLA-DMA, DLA-DQB1, DLA-DRA, HLA-DRB1, FOS, JAG2, and JUN) were involved in Th1-, Th2-, and Th17-cell differentiation in dogs with HF. The expression of DLA-DMA, DLA-DQB1, DLA-DRA, and HLA-DRB1 were upregulated in HF dogs (Figure 5B), whereas FOS, JAG2, and JUN were downregulated (Figure 5B).

### AS Detection

**Figure 6:**
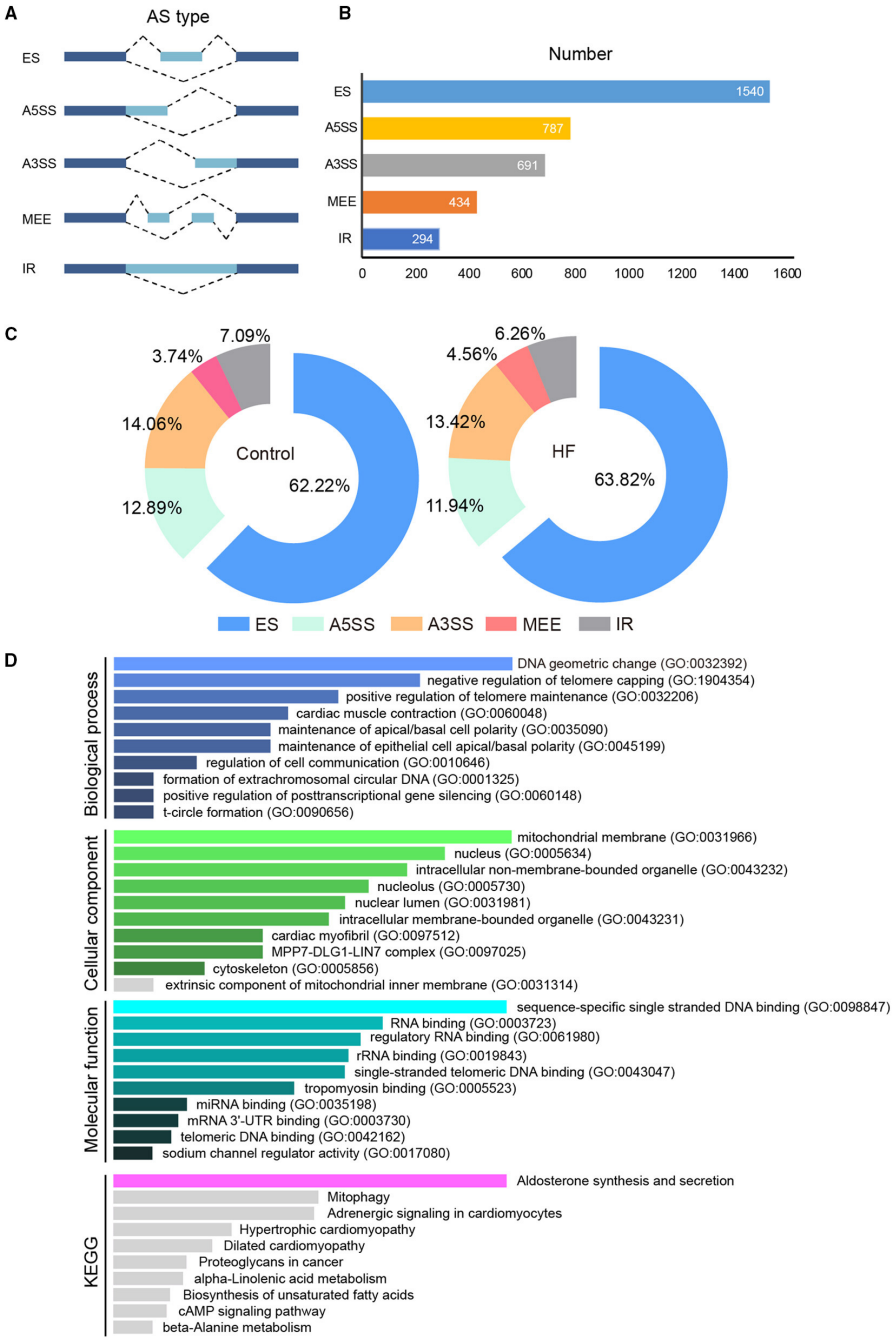
The alternative splicing analysis between HF dogs and controls. **(A)** Alternative splicing events. **(B)** Alternative splicing distribution. **(C)** The proportion of alternative splicing events. **(D)** Functional GO and KEGG analyses of the differentially alternative splicing genes between HF dogs and controls. Only the significant pathways sections (*p* < 0.05) were presented in the figure. AS, alternative splicing; A3SS, alternative 3′ splice site; ES, exon skipping; A5SS, alternative 5′ splice site; MEE, mutually exclusive exon; IR, intron retention; HF, heart failure; GO, Gene ontology; KEGG, Kyoto Encyclopedia of Genes and Genomes.

The full-length transcriptome sequencing can characterize the complexity of AS on a whole transcriptome scale. A total of 3,746 AS events were identified in both the HF and control groups. These events were classified into five categories (Figure 6A): (1) 1,540 (41%) exon skipping (ES), (2) 787 (21%) alternative 5′ splice site (A5SS), (3) 691 (18%) alternative 3′ splice site (A3SS), (4) 434 (12%) mutually exclusive exons (MEEs), and (5) 294 (8%) retained introns (RIs) (Figure 6B). Comparison of the AS events between the HF and control groups showed that the ES category occurred most frequently (control, 62.22%; HF, 63.82%), followed by A3SS (control, 14.06%; HF, 13.42%), A5SS (control, 14.06%; HF, 13.42%), IR (control, 7.09%; HF, 6.26%), and MEE (control, 3.74%; HF, 4.56%) (Figure 6C). Additionally, the AS analysis of the HF-related sarcomere genes revealed that five genes (*TTN, TNNI2, TNNI3, MYBPC3*, and *FLNC*) had variable splicing events (Table 3 and Supplementary File 2). The KEGG enrichment analysis revealed that differentially spliced genes were abundant in aldosterone synthesis and secretion, mitophagy, adrenergic signaling in cardiomyocytes, hypertrophic cardiomyopathy, and dilated cardiomyopathy (Figure 6D).

**Table 3:** Types of alternative splicing in the sarcomere genes in HF dogs.

**Gene symbol**	**Gene name**	**Alternative splicing type**
MYBPC3	Myosin-binding protein C, cardiac	A3SS, MES, SES
TTNI3	Troponin I type 3 (cardiac)	A5SS
TNNT2	Troponin T type 2 (cardiac)	A5SS, SES, A3SS, SES
TTN	Titin	MES, SES,A5SS
FLNC	Filamin C, gamma	SES

*A3SS, alternative 3′ splice site; A5SS, alternative 5′ splice site; MEE, mutually exclusive exon; MES, multiple exon skipping; SES, single exon skipping*.

### Transcription Factors and lncRNAs Analysis

**Figure 7:**
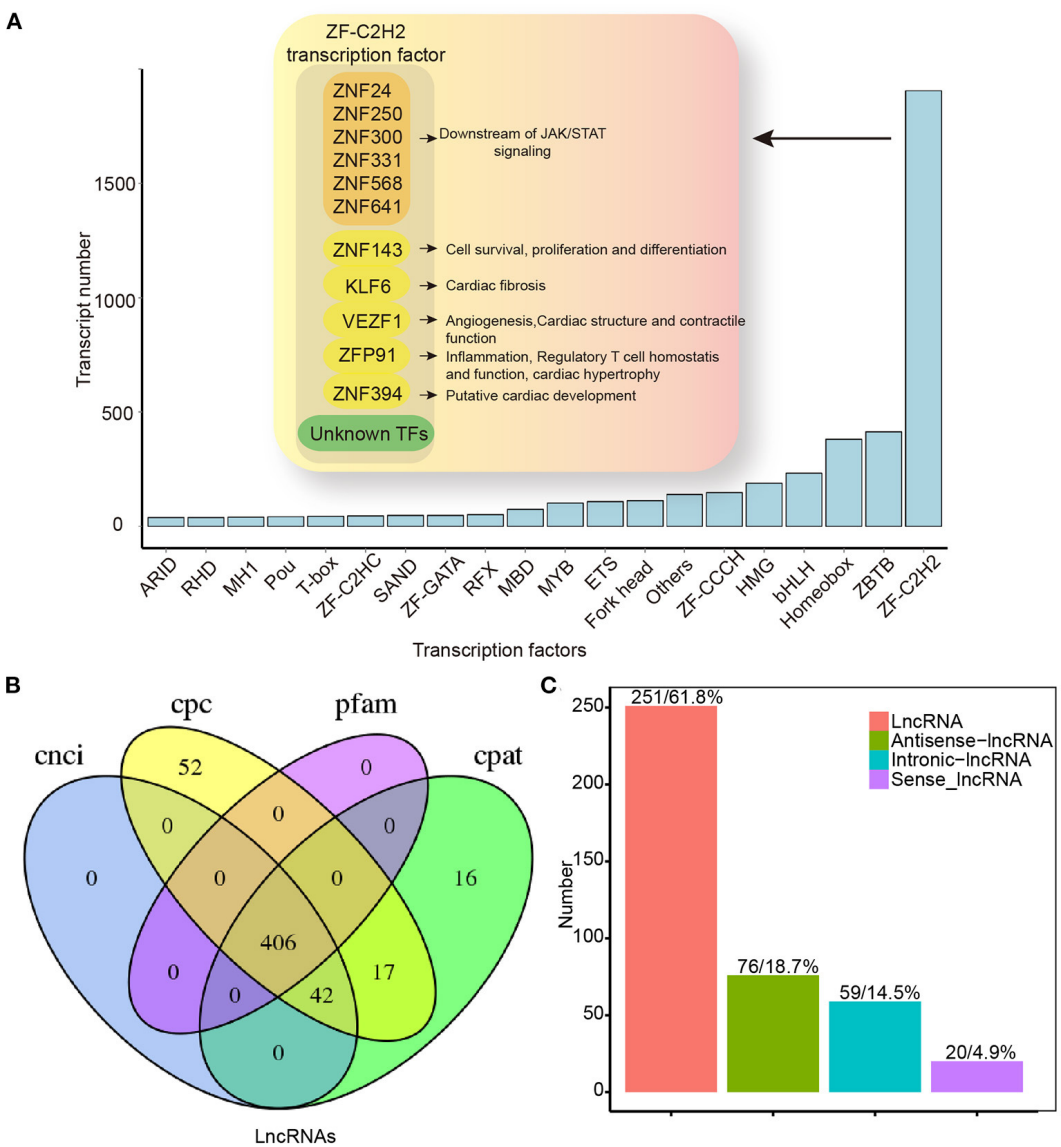
Transcription factors and lncRNAs analysis by full-length transcriptome sequencing. **(A)** TF distribution presenting information for only the top 20 TF families. **(B)** Venn map for the number of lncRNAs identified by CNCI, CPC, CPAT, and Pfam database. **(C)** LncRNAs positional classification. CPC, coding potential calculator; CNCI, coding–non-coding index; CPAT, coding potential assessment tool; CPM, counts per million; lncRNAs, long non-coding RNAs; TF, transcription factor.

In our study, 4,892 TFs from 62 different families were predicted using the AnimalTFDB 3.0 software^5^. Among them, ZF-C2H2, ZBTB, and Homeobox were abundant (Figure 7A). In the ZF-C2H2 group (the most abundant TF family), 1,905 transcripts corresponding to 1,426 TF genes were identified, including 17 alternative spliced genes (Figure 7A). For a better understanding of AS-associated biological processes, the functional characteristics are listed in Figure 7A. ZNF24, 250, 300, 331, 568, and 641 are known downstream effectors of JAK/STAT signaling by the KEGG analysis. Additionally, differentiated spliced TFs also function in cardiac fibrosis (e.g., KLF6) (19), angiogenesis (e.g., VEZF1) (20), cardiac structure and contractile function (e.g., VEZF1) (21), cardiac hypertrophy, inflammation, and regulatory T-cell homeostasis (e.g., ZFP91) (22–24) (Figure 7A). As critical effectors or regulators, TFs can sense multiple signal transduction pathways and metabolic perturbations in cells. In this study, up- and downregulated TF transcripts were analyzed, suggesting that differentially expressed TFs in the HF model were enriched in multiple signaling pathways. Upregulated TFs were mainly active in TGF-β signaling, ubiquitin-mediated proteolysis, and signaling pathways regulating stem cell pluripotency. Downregulated TFs, on the other hand, function in inflammatory bowel disease, acute myeloid leukemia, adipocytokine signaling pathway, etc. (Supplementary Figure 3).

In addition to protein-encoding transcripts, we identified lncRNAs. We predicted lncRNAs using the CNCI, CPC, CPAT, and Pfam database. A Venn diagram was used to represent the results of the four screening methods. As a result, 406 lncRNAs were identified (Figure 7B), and these lncRNAs were classified according to their location in the reference genome (Figure 7C). Except for binding activities with molecules (e.g., nucleic acids and proteins) (25), lncRNA functions diverged in the HF model. Upregulated lncRNAs were enriched mainly in cell metabolism (e.g., sugar and fatty acids), whereas downregulated genes were primarily enriched in RNA-mediated events (e.g., RNA processing, transport, and RNA-modulated diseases; Supplementary Figure 4).

## Discussion

Large animal models are an essential step in the development of therapeutics for HF (26, 27). Full-length transcriptome analysis based on large animals (dogs) was performed to elucidate more valuable clues regarding HF therapies. In a previous study, transcriptome analysis of a dog model of HF (especially rapid pacing HF) was performed using microarray (Table 4). Despite RNA sample testing using microarray technology being relatively precise for transcript quantitative analysis and customized, it often overlooks annotated and unidentified transcripts (including AS transcripts) outside the scope of testing (37). In our work, full-length transcript sequencing (nanopore platform) was used for data harvesting, with the aim of identifying more known and unknown transcripts and AS events. Compared to NGS, full-length transcriptome sequencing and its corresponding platform can deliver long reads that enable the precise construction of full-length splice variants. The read length (average reads length 1,153 bp) (Supplementary Table 1) contained in our study was longer than that obtained by NGS in a large animal model of HF (e.g., dog model of HF, average reads length 93 bp) (38). Recently, full-length transcript sequencing was also used in a pig model of HF (7). Our work focused on the identification of lncRNAs and AS events, and GO and KEGG analyses of DETs, providing more detailed information on HF research.

**Table 4:** Studies on transcriptome analysis in large animal model of HF.

**References**	**Species**	**Sample**	**Model**	**Platfdnn**	**Accession**
Ojaimi et al. (28)	Mongrel dog	Left ventricular tmyocardium	Pacing induced HF	Microarray	GSE5247
Gao et al. (29)	Dog	Left ventricular myocardium	Pacing induced HF	Microarray	GSE5247 GSE9794
Barth et al. (30)	Mongrel dog	Left ventricular myocardium	Tachypacing-induced HF (left bundle branch ablation + rapid atrial pacing)	Microarray	GSE5274 GSE9794 GSE14372 GSE14338 GSE14661
Lichter et al. (31)	Mongrel dog	Left ventricular myocardium	Tacltypacing-Induced HF (left bundle branch ablation + rapid atrial pacing)	Microarray	GSE14327 GSE14338
Wong et al. (32)	Sheep	Left ventricular myocardium	Pacing induced HF	RNA-seq, Illumina MicroRNA array	GSE87449
Tan et al. (6)	Minipig	Cardiac tissue	HEpEF (descending aortic constriction)	RNA-seq, Illumina	GSE143288
Vikholm et al. (33)	Pig	Right ventricular myocardium	Right ventricular HF (pulmonary banding)	Microarray	GSE31619
Argenziano et al. (34)	Dog	Right atrial ventricular myocardium	Pacing induced HF	Microarray	GSE12823
Torrado et al. (35, 36)	Pig	Cardiac tissue	HF (doxorubicin)	Microarray	GSE30110
Müller et al. (7)	German landrasse Pig	Left ventricular tissue	HF (post-infarction)	Nanopore RNA-seq, Illumina	NA
This study	Beagle	Right ventricular myocardium	Pacing induced HF	Nanopore	PRJNA731299

### Immune Responses Involved in the Development of HF

Functional antibodies and T-cell-mediated immunity, particularly Th1/Th17 responses, are active in HF-associated processes (39). The Th1/Th2 cytokine imbalance has also been attributed to the HF pathogenesis (40). Groschel et al. (39) demonstrated that T-helper cells specific for cardiomyocyte antigens could directly contribute to HF development independent of the autoantibodies. Compared to the control group, Th1, Th2, and Th17 populations increased in the HF model (41). Likewise, we found that Th1 and Th17 cells increased in patients with HF (Figure 5), indicating that immune responses indeed occurred in HF.

Dynamic changes occur in Th1/Th17 responses during HF (42). In this study, we found that Th1/Th17 differentiation-related DETs (Figure 4) and corresponding genes (e.g., *DLA-DMA, DLA-DQB1, DLA-DRA, HLA-DRB1, FOS, JAG2*, and *JUN*) were differentially expressed (Figure 5). It has been reported that endothelin 1, fibronectin, TGF-β, and collagen deposition are induced by the JUN–FOS module (43, 44). JAG2, a critical ligand in Notch signaling, modulates cell differentiation, cell elongation, and cell death and functions in Treg/Th17 differentiation (45, 46). In addition, DLA-DMA, DLA-DQB1, DLA-DRA, and HLA-DRB1 are indispensable for CD4^+^ T-cell activation and Th1/Th17 induction (47, 48). Even though the enhancement of Th1/Th17 differentiation by HF has been confirmed, the inner mechanism by which cardiomyocytes initiate and activate immune cell differentiation remains unclear. Overall, our findings raise the possibility that the underlying mechanisms of Th1/Th17 modulation might be a clue on how the immune system functions in HF.

### Implications of AS in HF

Alternative splicing is an important post-transcriptional regulatory mechanism that exists widely in living organisms (49). Various AS types are responsive to HF (50, 51). For example, AS modulates the expression of sarcomeric genes in heart diseases (49, 52, 53), and dysfunctional myocardial cells may accumulate by aberrant splicing. Sarcomeric genes such as *TTN, TNNI3, TNNT2*, and *MYH7* are closely associated with HF (51). Moreover, the AS frequency in the *TTN* gene increases in HF, meaning it could potentially serve as an indicator of HF (51). Except for *TTN*, we found multiple AS events of *TNNI3, TNNT2*, and *FLNC* genes in HF (Supplementary File 2), implying that AS event occurrence might be more complicated than imagined. HF may share similar AS events with other cardiovascular diseases. Because of the harvesting of more information by full-length transcriptome sequencing, novel AS events were identified in this study. For example, for the first time in HF research, MYBPC3 was differentially spliced. Furthermore, AS-related genes analyzed by GO and KEGG analyses were involved in heart diseases (Figure 6). This supports the suggestion that AS events may be helpful in HF diagnosis and prognostic marker digging.

### Analysis of the Transcription Factors and lncRNAs in HF

Transcription factors are proteins that bind DNA regulatory sequences for enhancing or silencing gene transcription (54). In complex cell networking, TFs act downstream of multiple signal transduction pathways (e.g., immune-associated pathways) and selectively modulate effector gene expression (55). Digging deep into changes in TF could provide a better understanding of HF responses. In our study, the ZF-C2H2 group was highly enriched in HF (Figure 7), some of which underwent variable splicing events and participated in cardiac remodeling, immune inflammation, myocardial contraction, and the JAK/STAT signaling pathway (21, 22, 56). This indicates that the differentially expressed TFs are closely related to HF. On the one hand, we found that upregulated TFs were mainly enriched in TGF-β signaling that modulated myocardial fibrosis (Supplementary Figure 3). On the other hand, downregulated genes were involved in immune- and inflammation-related diseases (Supplementary Figure 3), whether such diseases and HF share signaling pathways remains unknown.

The lncRNAs are novel regulators of cardiovascular diseases (57). In the past 10 years, lncRNAs have been extensively identified and annotated in detail (58). Moreover, many studies have found that lncRNAs participate in cardiomyocyte metabolism by regulating gene transcription and maintaining the homeostasis of cardiomyocytes (58, 59). In our study, differentiated lncRNAs were involved in cell metabolism, protein, and RNA processing pathways, such as the pentose phosphate pathway and lysosome modulation (Supplementary Figure 4), suggesting that HF might be widely influenced by lncRNA. Overall, full-length transcriptome sequencing offers additional clues for understanding HF.

### Limitations

This study had several limitations. First, the sequencing capacity of the nanopore technique may not have been adequate to cover the entire length of the involved genes. Therefore, genes with low expression levels may have been missed in our analysis. Second, although we validated the significance of the sequencing results using molecular experiments, no in-depth characterization of the transcript variant landscape was achieved. Thus, the impact of TFs and lncRNAs on HF should be studied in more detail.

### Conclusion

Our full-length transcriptome sequencing of myocardial tissues from HF dogs improved our understanding of transcriptome diversity. Consequently, understanding the characteristics associated with the biological phenotype of HF and the potential intervention target genes may pave the way for improved treatment of HF.

## Data Availability Statement

The datasets presented in this study can be found in online repositories. The names of the repository/repositories and accession number(s) can be found at: NCBI SRA; PRJNA731299.

## Ethics Statement

The studies involving human participants were reviewed and approved by the Ethics Committee of the First Affiliated Hospital of Xinjiang Medical University. The patients/participants provided their written informed consent to participate in this study. The animal study was reviewed and approved by Animal Welfare Authority and Ethics Committee of the First Affiliated Hospital of Xinjiang Medical University.

## Author Contributions

XL, ZB, and FW wrote the manuscript. XZ, ZB, YL, and BT contributed to the funding acquisition, conception, and design of the study. HS, YH, and JX contributed to the animal experiments. XL, ZB, XZ, and JX contributed to the statistical analysis and data interpretation. All authors approved the submitted version.

## Funding

This study was supported by the National Natural Science Foundation of China (project no: 81860081).

## Conflict of Interest

The authors declare that the research was conducted in the absence of any commercial or financial relationships that could be construed as a potential conflict of interest.

## Publisher's Note

All claims expressed in this article are solely those of the authors and do not necessarily represent those of their affiliated organizations, or those of the publisher, the editors and the reviewers. Any product that may be evaluated in this article, or claim that may be made by its manufacturer, is not guaranteed or endorsed by the publisher.

## Footnotes

^1^ https://github.com/lh3/minimap2

^2^ http://genome.imim.es/astalavista

^3^ http://kobas.cbi.pku.edu.cn

^4^ https://maayanlab.cloud/Enrichr/

^5^ http://bioinfo.life.hust.edu.cn/AnimalTFDB/

## Supplementary Material

Supplementary Figure 1 **(A)** Category of cellular components and molecular functions in GO annotation for upregulated differentially expressed transcripts (DETs). **(B)** Category of cellular components and molecular functions in GO annotation for downregulated differentially expressed transcripts (DETs). Only the significant pathways sections (*p* < 0.05) are shown.

Supplementary Figure 2 **(A)** Volcano plots of differentially expressed genes. Green represents downregulated differentially expressed genes, and red represents upregulated differentially expressed genes. **(B)** Gene Ontology (GO) and Kyoto Encyclopedia of Genes and Genomes (KEGG) analysis of DEGs between HF and control groups. Only the significant pathways sections (*p* < 0.05) are shown. DEGs, Differentially expressed genes; FC, fold change.

Supplementary Figure 3 KEGG/GO analysis of differentially expressed transcription factors. Only the significant pathways sections (*p* < 0.05) are shown. TFs, Transcription factors.

Supplementary Figure 4 KEGG/GO analysis of differentially expressed lncRNAs. Only the significant pathways sections (*p* < 0.05) are shown. lncRNAs, long non-coding RNAs.

Supplementary Table 1 Data quality control information of full-length transcriptome sequencing.

Supplementary Table 2 Full-length sequence statistics of each sample.

Supplementary File 1 Differentially expressed transcripts in myocardial tissue from HF dogs and controls.

Supplementary File 2 Alternative splicing events of the sarcomeric genes in HF dogs.

## Abbreviations

HF: heart failure
BNP: B-type natriuretic peptide
TGS: third-generation sequencing
AS: alternative splicing
NGS: next-generation sequencing
ONT: Oxford Nanopore Technologies
CPC: coding potential calculator
CNCI: coding–non-coding index
CPAT: coding potential assessment tool
CPM: counts per million
DETs: differentially expressed transcripts
GO: Gene Ontology
KEGG: Kyoto Encyclopedia of Genes and Genomes
ES: exon skipping
A5SS: alternative 5′ splice site
A3SS: alternative 3′ splice site
MEEs: mutually exclusive exons
EF: ejection fraction
TFs: transcription factors
lncRNAs: long non-coding RNAs
RVP: rapid ventricular pacing
IQR: interquartile range
BMI: body mass index
TGF: transforming growth factor.
