# Supplementary figures and images for: Full-Length Transcriptome Sequencing: An Insight Into the Dog Model of Heart Failure
Source: Front Cardiovasc Med. 2021 Dec 16;8:712797. doi: 10.3389/fcvm.2021.712797 (PMC8716442; doi:10.3389/fcvm.2021.712797)

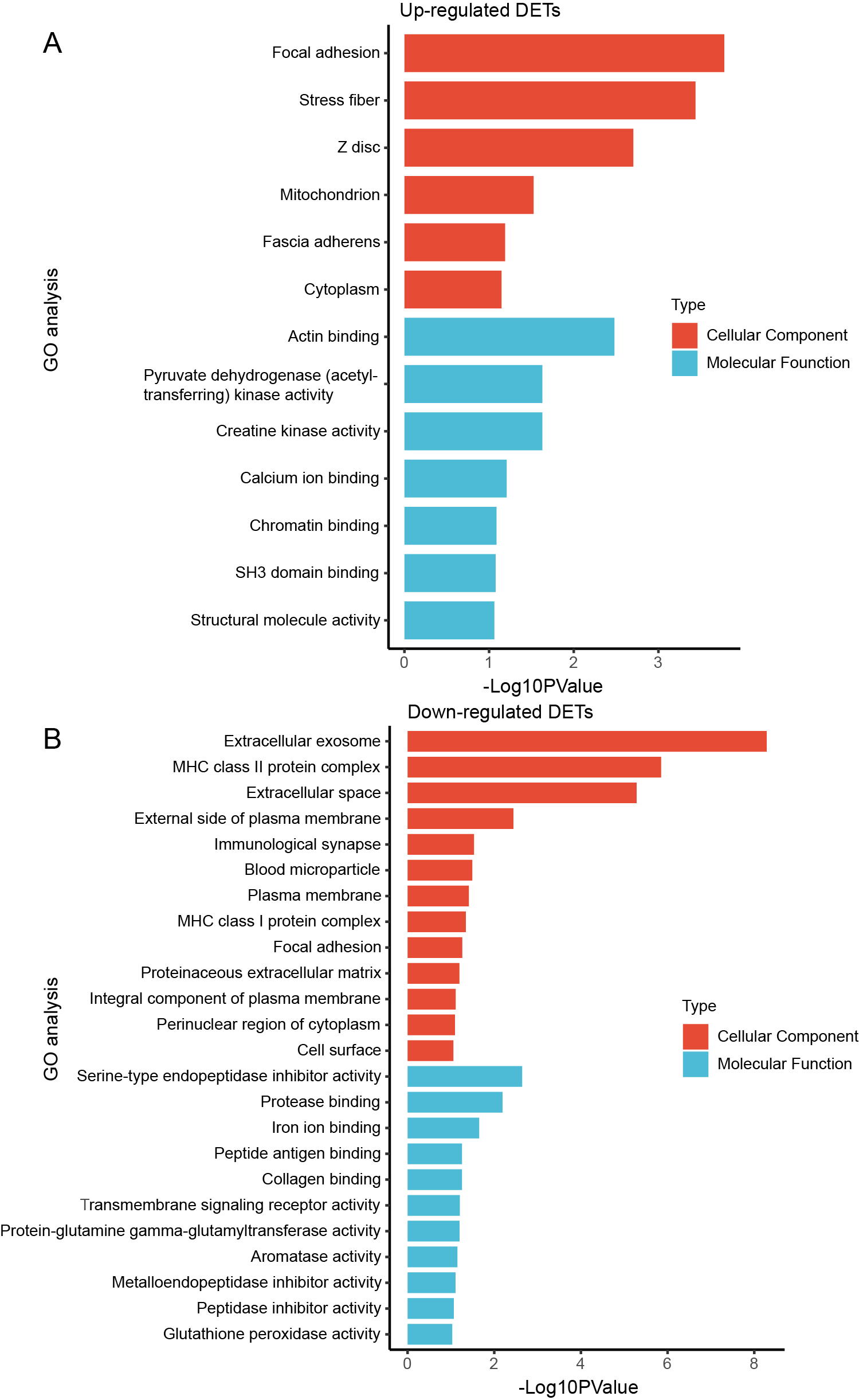

Supplement: Supplementary Figure 1 — (A) Category of cellular components and molecular functions in GO annotation for upregulated differentially expressed transcripts (DETs). (B) Category of cellular components and molecular functions in GO annotation for downregulated differentially expressed transcripts (DETs). Only the significant pathways sections (p < 0.05) are shown. [file Image_1.TIF]

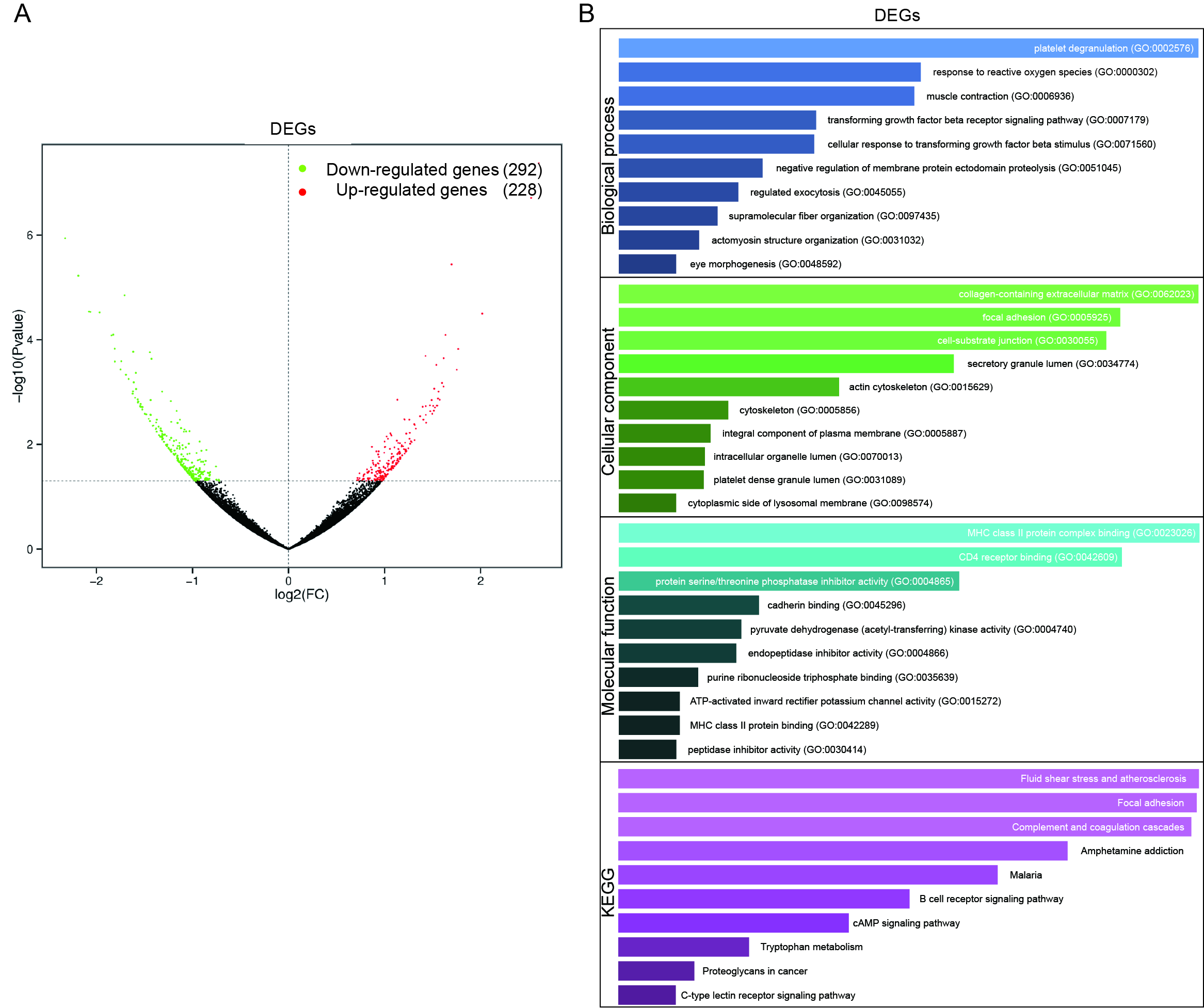

Supplement: Supplementary Figure 2 — (A) Volcano plots of differentially expressed genes. Green represents downregulated differentially expressed genes, and red represents upregulated differentially expressed genes. (B) Gene Ontology (GO) and Kyoto Encyclopedia of Genes and Genomes (KEGG) analysis of DEGs between HF and control groups. Only the significant pathways sections (p < 0.05) are shown. DEGs, Differentially expressed genes; FC, fold change. [file Image_2.TIF]

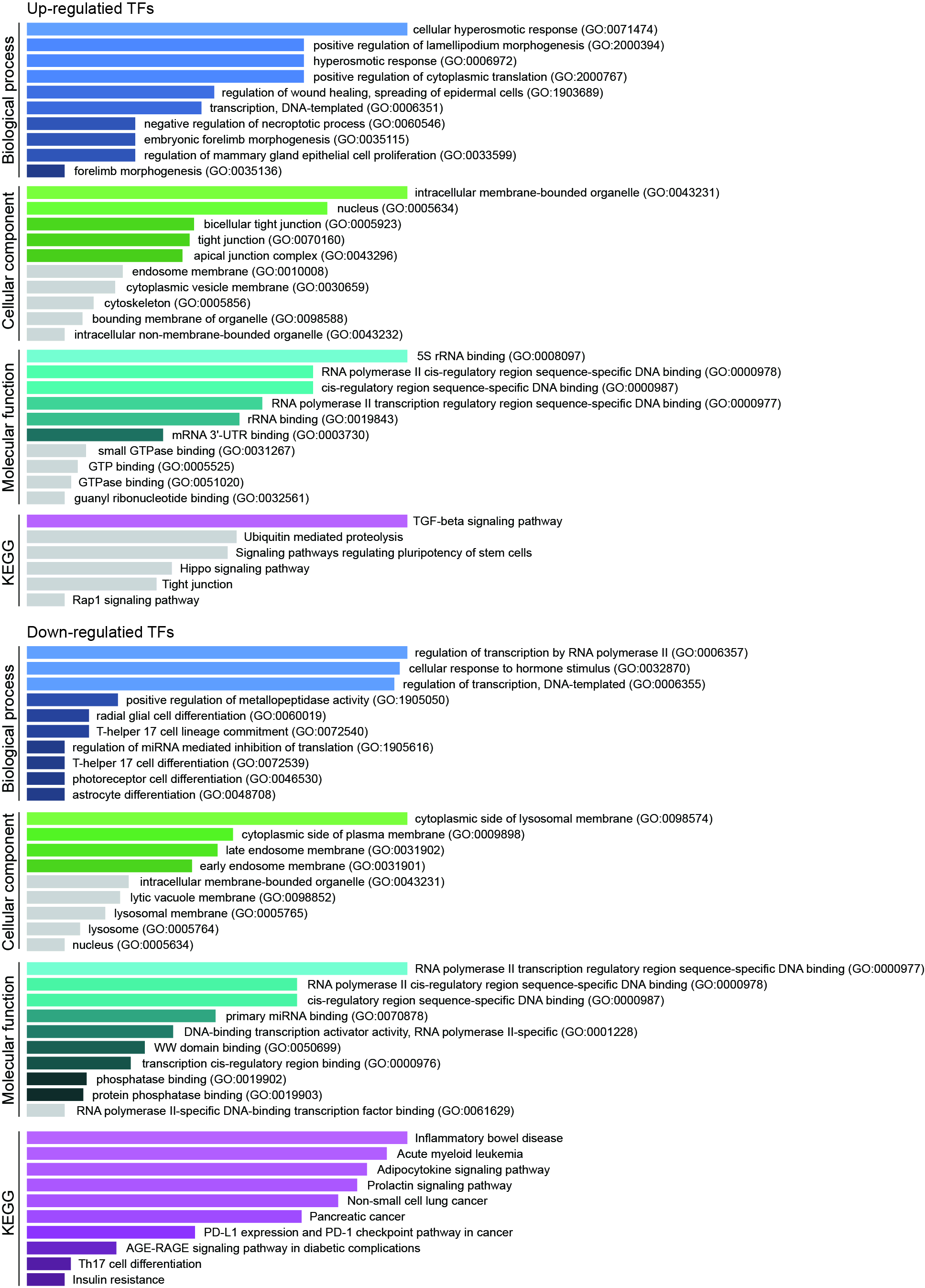

Supplement: Supplementary Figure 3 — KEGG/GO analysis of differentially expressed transcription factors. Only the significant pathways sections (p < 0.05) are shown. TFs, Transcription factors. [file Image_3.TIF]

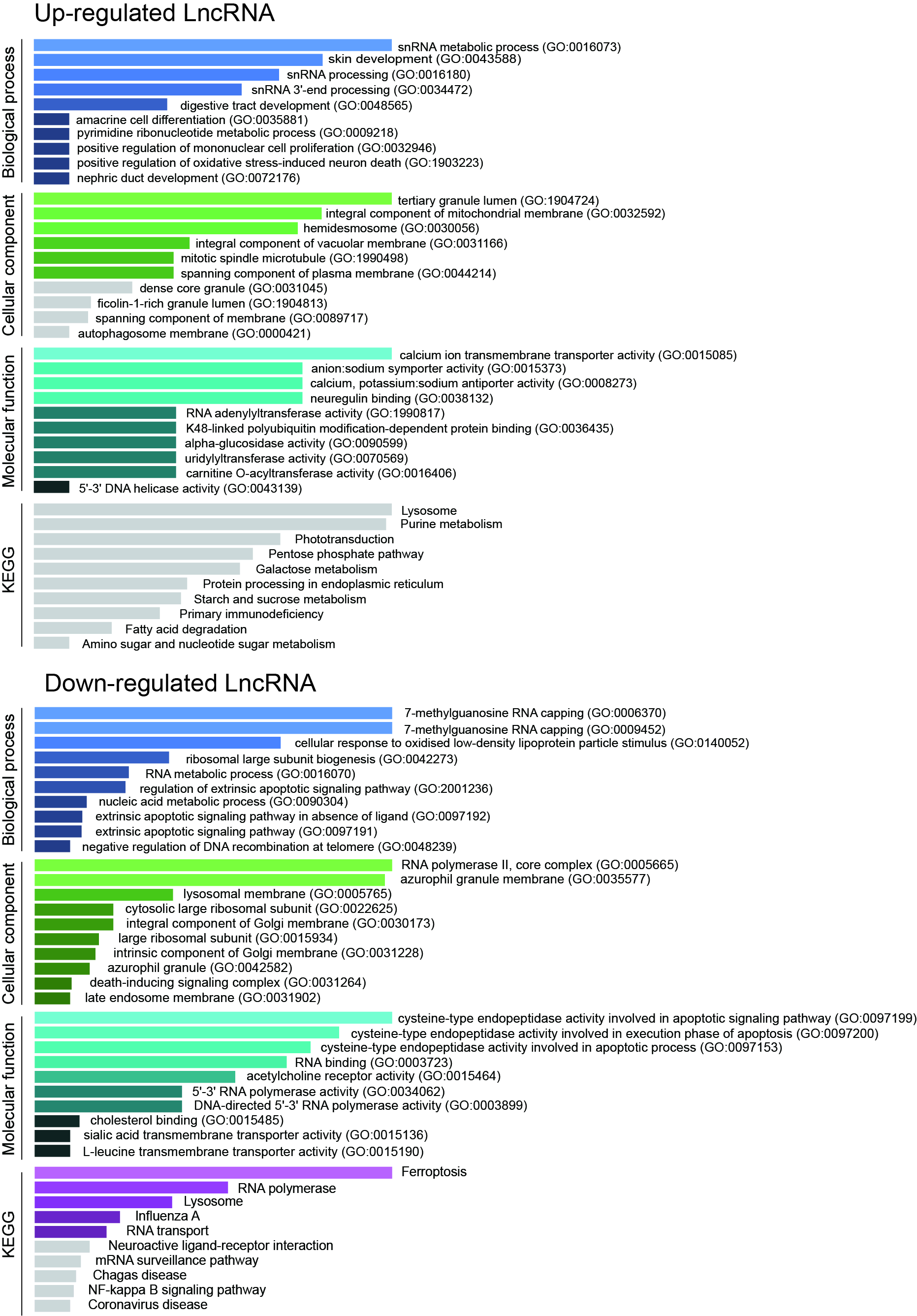

Supplement: Supplementary Figure 4 — KEGG/GO analysis of differentially expressed lncRNAs. Only the significant pathways sections (p < 0.05) are shown. lncRNAs, long non-coding RNAs. [file Image_4.TIF]
