# Supplementary material for: Full-Length Transcriptome Sequencing: An Insight Into the Dog Model of Heart Failure
Source: Front Cardiovasc Med. 2021 Dec 16;8:712797. doi: 10.3389/fcvm.2021.712797 (PMC8716442; doi:10.3389/fcvm.2021.712797)
Supplement: Supplementary Table 1 — Data quality control information of full-length transcriptome sequencing. [file Table_1.DOCX]

**Supplemental Table S1. Data quality control information of full-length transcriptome sequencing.**

| Sample | Read Number | Base  Number | N50  (bp) | Mean Length (bp) | Max  Length (bp) | Mean  Q score |
| --- | --- | --- | --- | --- | --- | --- |
| C1 | 1,545,030 | 1,660,729,476 | 1,165 | 1,074 | 255,138 | Q12 |
| C2 | 2,110,015 | 2,745,863,480 | 1,517 | 1,301 | 207,867 | Q12 |
| C3 | 2,147,057 | 2,706,012,650 | 1,454 | 1,260 | 67,538 | Q12 |
| HF1 | 1,838,434 | 2,242,222,532 | 1,382 | 1,219 | 90,995 | Q12 |
| HF2 | 1,856,032 | 2,116,781,159 | 1,311 | 1,140 | 473,251 | Q12 |
| HF3 | 2,422,693 | 2,249,621,625 | 950 | 928 | 261,619 | Q12 |

C：controls, HF: heart failure.
