# Supplementary material for: Full-Length Transcriptome Sequencing: An Insight Into the Dog Model of Heart Failure
Source: Front Cardiovasc Med. 2021 Dec 16;8:712797. doi: 10.3389/fcvm.2021.712797 (PMC8716442; doi:10.3389/fcvm.2021.712797)
Supplement: Supplementary Table 2 — Full-length sequence statistics of each sample. [file Table_2.DOCX]

| SampleID | Number of clean reads  (except rRNA) | Number of full-length reads | Full-Length Percentage (FL%) |
| --- | --- | --- | --- |
| C1 | 1105125 | 912160 | 82.54% |
| C2 | 1,614,183 | 1323524 | 81.99% |
| C3 | 1,691,556 | 1397903 | 82.64% |
| HF1 | 1,435,967 | 1223137 | 85.18% |
| HF2 | 1,416,072 | 1173507 | 82.87% |
| HF3 | 1,800,561 | 1565375 | 86.94% |

**Supplemental Table S2. Transcript full-length sequence statistics of each sample.**

C: control; HF: heart failure
